# Analytical Post-Embedding Immunogold–Electron Microscopy with Direct Gold-Labelled Monoclonal Primary Antibodies against RIBEYE A- and B-Domain Suggests a Refined Model of Synaptic Ribbon Assembly

**DOI:** 10.3390/ijms25137443

**Published:** 2024-07-06

**Authors:** Stella Papadopoulos, René Tinschert, Iason Papadopoulos, Xenia Gerloff, Frank Schmitz

**Affiliations:** 1 Institute of Anatomy, Department of Neuroanatomy, Medical School, Saarland University, 66421 Homburg, Germany; stella.papadopoulos@uks.eu (S.P.); tinschert.rene@gmail.com (R.T.); 2 Mathematical Institute, University of Bonn, 53115 Bonn, Germany; s17ipapa@uni-bonn.de (I.P.); s06xgerl@uni-bonn.de (X.G.)

**Keywords:** retina, synaptic ribbon, RIBEYE, RIBEYE A-domain, RIBEYE B-domain, immunogold electron microscopy, direct gold-labelled primary antibodies

## Abstract

Synaptic ribbons are the eponymous specializations of continuously active ribbon synapses. They are primarily composed of the RIBEYE protein that consists of a unique amino-terminal A-domain and carboxy-terminal B-domain that is largely identical to the ubiquitously expressed transcriptional regulator protein CtBP2. Both RIBEYE A-domain and RIBEYE B-domain are essential for the assembly of the synaptic ribbon, as shown by previous analyses of RIBEYE knockout and knockin mice and related investigations. How exactly the synaptic ribbon is assembled from RIBEYE subunits is not yet clear. To achieve further insights into the architecture of the synaptic ribbon, we performed analytical post-embedding immunogold–electron microscopy with direct gold-labelled primary antibodies against RIBEYE A-domain and RIBEYE B-domain for improved ultrastructural resolution. With direct gold-labelled monoclonal antibodies against RIBEYE A-domain and RIBEYE B-domain, we found that both domains show a very similar localization within the synaptic ribbon of mouse photoreceptor synapses, with no obvious differential gradient between the centre and surface of the synaptic ribbon. These data favour a model of the architecture of the synaptic ribbon in which the RIBEYE A-domain and RIBEYE B-domain are located similar distances from the midline of the synaptic ribbon.

## 1. Introduction

Ribbon synapses are continuously active synapses in the retina, inner ear, and pineal gland [1,2,3]. The continuous activity of ribbon synapses requires structural, functional, and molecular specializations. The synaptic ribbon is the most prominent structural specialization of this synapse. It is a large electron-dense structure that associates with the presynaptic active zone. The synaptic ribbons can adopt various three-dimensional shapes in the different types of ribbon synapses but always bind large numbers of synaptic vesicles, which are delivered to the active zone to foster continuous synaptic vesicle exocytosis [4,5,6].

RIBEYE is the main protein component of synaptic ribbons and consists of two major protein domains: an amino-terminal proline-rich A-domain (amino acids (aa)1-aa563 in mice) and a carboxy-terminal NAD(H)-binding B-domain (aa564-aa988 in mice) [7,8,9]. The A-domain of RIBEYE (aa1-aa563 in mice) is unique to RIBEYE. In contrast, the B-domain of RIBEYE is largely identical to the transcriptional co-repressor CtBP2 [7]. Only the first 20 amino-terminal amino acids of CtBP2, which contain a nuclear localization signal, are lacking from the RIBEYE B-domain. *RIBEYE* and *CtBP2* transcripts are generated from the same bifunctional *RIBEYE/CtBP2* gene by using different promoters [7,10]. Remarkably, RIBEYE is only expressed in ribbon synapses [3,7], whereas CtBP2 is nearly ubiquitously expressed [11]. CtBP2 and CtBP1 are highly homologous NAD(H)-binding transcriptional regulators in the nucleus and developed from a family of D isomer-specific 2-hydroxyacid dehydrogenases [12].

The analysis of RIBEYE knockout mice with a deletion of the exon encoding RIBEYE A-domain, together with data from cell transfection experiments, suggested that RIBEYE A-domain is essential for the assembly of synaptic ribbons [7,9,13,14,15,16,17]. In agreement with the central role of the RIBEYE A-domain for the assembly of synaptic ribbons, RIBEYE contains multiple interaction sites, particularly in the RIBEYE A-domain, which can interact with other RIBEYE molecules [17]. Multiple RIBEYE-RIBEYE interactions are likely required to form a large three-dimensional structure, such as the synaptic ribbon. However, it is not yet clear how the synaptic ribbon can assemble from individual RIBEYE protein subunits.

Since the A-domain is unique to RIBEYE it has been proposed that the A-domain has a predominantly structural role in the assembly of the synaptic ribbon [7]. But recently, it became clear that the RIBEYE B-domain is also essential for synaptic ribbon assembly and is also needed to build the synaptic ribbon [15].

Clearly, more data are needed to better understand the building of synaptic ribbons and their assembly from RIBEYE subunits. In the present study, we aimed to obtain further insights into the architecture of the synaptic ribbon. We used well-characterized monoclonal antibodies against the RIBEYE A-domain and RIBEYE B-domain that were directly labelled with colloidal gold particles to obtain an improved ultrastructural resolution on the localization of these protein domains within the synaptic ribbon by using post-embedding immunogold electron microscopy.

For our analyses, we focused on rod photoreceptor ribbon synapses of the mouse retina. The mouse retina is a rod photoreceptor-dominated retina. More than 95% of photoreceptor synapses are rod photoreceptor synapses. Rod photoreceptor synapses establish a fairly homogenous synapse population with a uniform morphology [3]. They contain a single active zone with a single large synaptic ribbon. In cross-sectioned rod photoreceptor synapses, the synaptic ribbon typically appears as a bar-shaped structure (width of ~30–40 nm, several 100 nm in height in transmission electron microscopy, TEM). Lateral/tangential views of the synaptic ribbon and/or serial TEM sections/tomography reveal the plate-like three-dimensional structure of rod photoreceptor synaptic ribbons [18]. The ribbon can be more than 1.5 μm in length in the z-direction [3].

## 2. Results

We performed post-embedding immunogold microscopy with direct gold-labelled primary antibodies against the RIBEYE A-domain (clone 6F4) [15] and RIBEYE B-domain (clone 2D9) [15,19] to obtain more detailed information on the localization of these protein domains within the synaptic ribbon. Previously, we showed that these antibodies were suitable for post-embedding immunogold labelling of the synaptic ribbon with the “indirect” immunogold technique [15,19]. This indirect immunolabelling technique applies gold-labelled secondary antibodies to detect the binding of the unlabelled primary antibodies. Inevitably, the indirect labelling procedure has a lower spatial resolution than the direct immunolabelling technique because primary and secondary antibodies are required for antigen localization, which obviously enlarges the distance between a gold particle and the primary antibody’s epitope.

**Figure 1:**
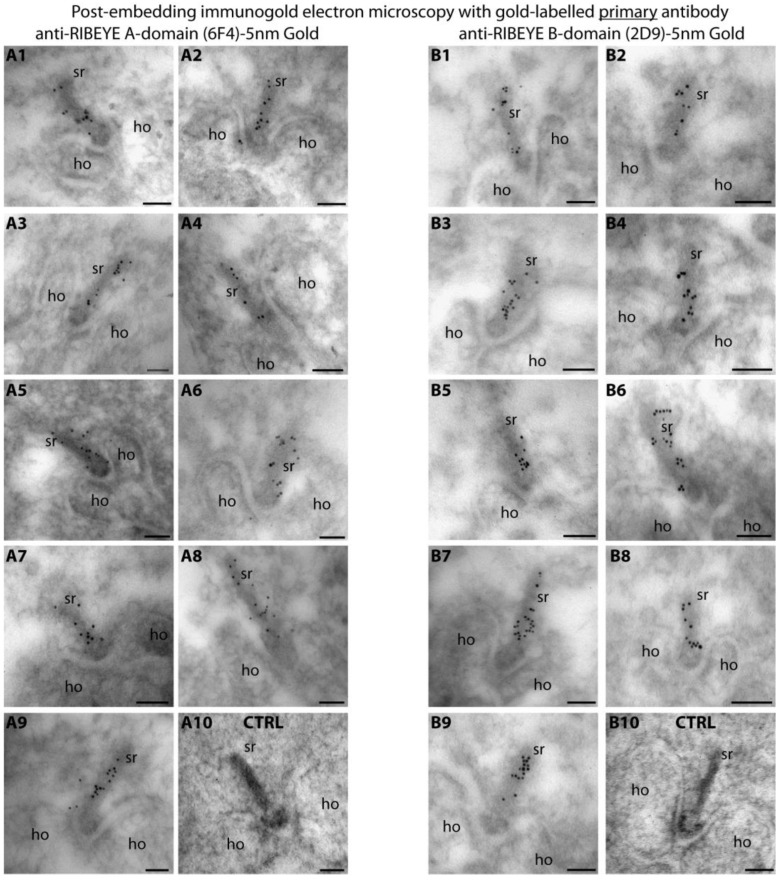
Post-embedding immunolabelling with gold-conjugated primary antibodies against RIBEYE A-domain (6F4) and RIBEYE B-domain (2D9). Ultrathin LR Gold sections of the mouse retina were incubated with gold-labelled primary antibodies against the RIBEYE A-domain (6F4) (**A1**–**A9**) or RIBEYE B-domain (**B1**–**B9**). (**A10**,**B10**) show the results of negative control experiments in which the retina sections were incubated only with gold colloid (without conjugation to primary antibody). Abbreviations: sr, synaptic ribbon; ho, horizontal cell dendrite; CTRL, negative control incubation. Scale bars: 50 nm.

Therefore, we used direct labelled primary antibodies to obtain a better resolution on the distribution of the RIBEYE A-domain and RIBEYE B-domain within the synaptic ribbon in the present study (Figure 1). Both gold-labelled primary RIBEYE antibodies produced qualitatively identical immunolabelling results (Figure 1), as previously observed with the indirect immunogold labelling with these antibodies [15,19]. The synaptic ribbon was strongly immunolabelled with direct gold-labelled primary antibody against the RIBEYE A-domain (Figure 1(A1–A9)) and direct gold-labelled primary antibody against the RIBEYE B-domain (Figure 1(B1–B9)). The specific immunolabelling of synaptic ribbons with the gold-labelled primary RIBEYE antibodies demonstrated that the primary antibodies retained their activity and specificity after conjugation with the colloidal gold particles. Negative control experiments did not show labelling of the synaptic ribbon (Figure 1(A10,B10)).

**Figure 2:**
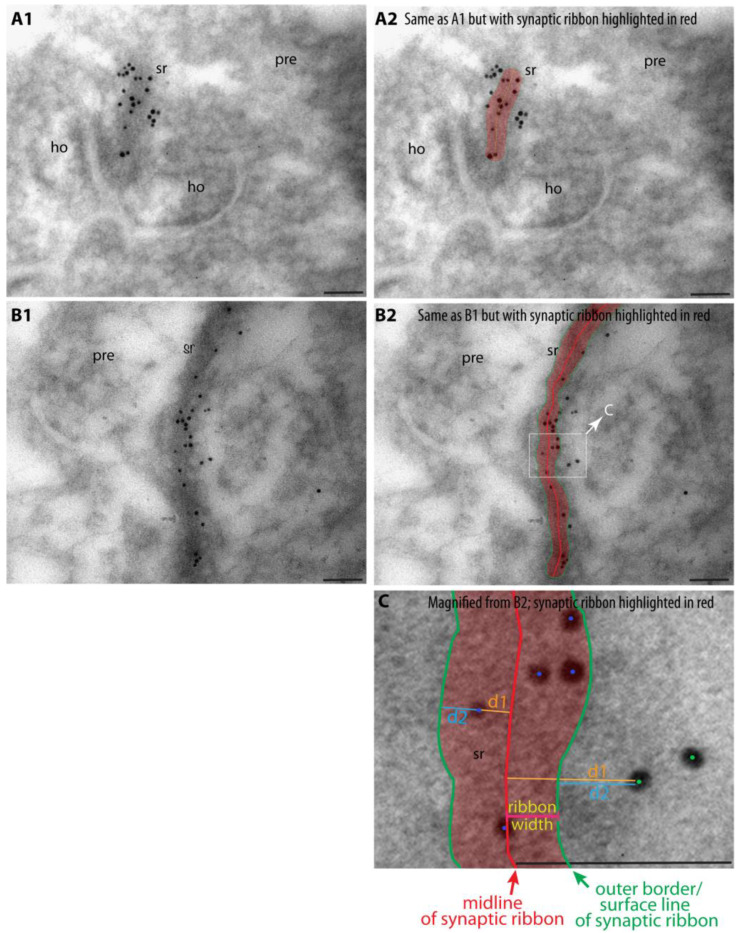
This figure summarizes central methodological steps of how we determined the localization of immunogold puncta with reference to the midline of the synaptic ribbon. Exemplary LR Gold ultrathin sections from the mouse retina immunolabelled with antibodies against RIBEYE A-domain and RIBEYE B-domain. Images from (**A1**,**A2**,**B1**,**B2**) were acquired at a magnification of 135,000×. Immunolabelled synaptic ribbons, as shown in (**B2**), were electronically magnified to a final magnification of ~788,000× (**C**) for measuring the relative distance (d_rel_) to the ribbon midline. d_rel_ was calculated according to Equation (1) for immunogold puncta located within the synaptic ribbon and with Equation (2) for puncta outside of the synaptic ribbon. Blue dots denote exemplary gold particles located within the synaptic ribbon; green dots denote exemplary gold particles located outside of the synaptic ribbon. Abbreviations: d1, minimal distance between midline of synaptic ribbon and gold particle; d2, minimal distance between gold particle and outer border/surface line of synaptic ribbon; sr, synaptic ribbon; pre, presynaptic, ho, horizontal cell dendrite. Scale bars: 50 nm.

For the quantitative analyses of the localization of the RIBEYE A-domain and RIBEYE B-domain immunogold puncta, the automated analysis programme calculated the shortest distance of each individual immunogold particle both to the centre of the ribbon (value denoted as d1, shown as exemplary orange lines in Figure 2C) as well as to the outer border of the synaptic ribbon (denoted as d2, shown as exemplary blue lines in Figure 2C). Next, the d1 and d2 values were used to determine the normalized relative distance (d_rel_) of the individual immunogold puncta from the centre of the synaptic ribbon according to Equations (1) and (2) (see Section 4). Immunogold particles could be located directly on the synaptic ribbon or outside of the synaptic ribbon. Equation (1) (see Section 4) was used to calculate the d_rel_ values for gold particles within the synaptic ribbon; Equation (2) (see Section 4) was used for gold particles outside of the synaptic ribbon. In this way, the absolute distance of the individual gold particles to the centre of the synaptic ribbon is normalized for both ribbon-associated and non-ribbon-associated gold particles to the individual width of the synaptic ribbon.

The relative distance (d_rel_) obtained using this way is more meaningful than the absolute distance of gold puncta (in nm) from the synaptic ribbon midline. The synaptic ribbon is a large three-dimensional structure, and the apparent width of the synaptic ribbon (in nm) thus strongly depends on the section angle. The relative distance (d_rel_) values obtained in this way are values that are corrected/normalized to the individual thickness of the analyzed synaptic ribbon.

For immunogold puncta located inside the synaptic ribbon, relative distance (d_rel_) values range from 0 (location of immunogold puncta on the ribbon midline) to 1 (location on the outer border/surface of the synaptic ribbon). 

For RIBEYE immunogold puncta outside of the synaptic ribbon, the relative distance (d_rel_) values are larger >1, and are larger the more distant they are located from the surface of the synaptic ribbon.

**Figure 3:**
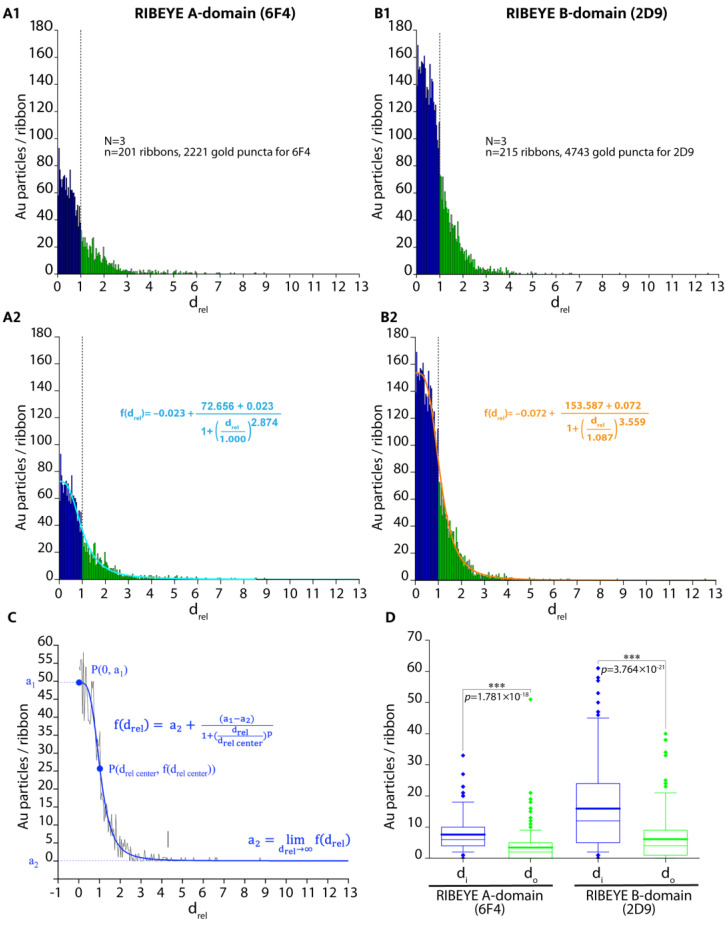
Relative distance (d_rel_) values for the RIBEYE A-domain and RIBEYE B-domain immunogold puncta were plotted. In blue, immunogold puncta with d_rel_ values ≤1, i.e., immunogold puncta located directly on the synaptic ribbon, are highlighted. In green, immunogold puncta with d_rel_ > 1, i.e., located outside of the synaptic ribbon (as defined by Equation (2)), are highlighted (**A1**,**A2**,**B1**,**B2**). (**A2**,**B2**) are identical with (**A1**,**B1**) except that a logistic fit curve (highlighted in blue and orange) was added that fits best the distribution of the d_rel_ values of RIBEYE A-domain and RIBEYE B-domain immunogold puncta. The dotted lines in (**A1,A2,B1,B2**) separate immunogold puncta with d_rel_ values ≤ 1 from immunogold puncta with d_rel_ > 1. (**C**) schematically shows by an example the definition of parameters of the logistic curve that we used to fit the experimental data from (**A2**,**B2**). Curve parameters for the fit curve in (**A2**) (numbers are rounded): a_1_: 72.656, a_2_: −0.023; d_rel center_: 1.000; p: 2.874; curve parameters for the fit curve in (**B2**) (numbers are rounded): a_1_: 153.587, a_2_: −0.072, d_rel center_: 1.087, p: 3.5594. In (**D**) the number of immunogold particles directly on the synaptic ribbon (d_i_) and outside of the synaptic ribbon area (d_o_) were plotted for direct labelled antibodies for RIBEYE A-domain (6F4) and RIBEYE B-domain (2D9). Boxes represent the 25th to 75th percentiles of datapoints (interquartile range (Q1–Q3)), the lower whisker the 0.05-quantile (5% quantile) and the upper whisker the 0.95-quantile (95% quantile). Thick lines depict the arithmetic mean values, amd thin represent lines of the median values. Lower diamonds are datapoints smaller than the 0.05-quantile; upper diamonds are datapoints larger than the 0.95-quantile. Abbreviations: P(x,y), punctum with x/y coordinates; f, function value; p, power; d_rel_, relative distance; ***, *p* ≤ 0.001.

For quantitative analyses, the relative distance (d_rel_) data obtained with the directly labelled primary antibodies were plotted with OriginPro (Figure 3(A1,B1)) for the RIBEYE A-domain and RIBEYE B-domain immunogold puncta. Data were obtained from three independent immunolabelling experiments from three different embeddings (Figure 3(A1,A2,B1,B2)).

In Figure 3(A2,B2), a fit curve was added to the d_rel_ raw data of the RIBEYE A-domain and RIBEYE B-domain to extract and analyze quantitative values from the data (Figure 3). A logistic fit was found to fit best the data following the equation for f(d_rel_) (Figure 3C). Quantitative plotting of the relative distance (d_rel_) values shown in Figure 3 demonstrated that the majority of immunogold puncta were located on the synaptic ribbons in the case of post-embedding immunogold labelling, i.e., they show relative distance (d_rel_) values between 0 and 1 (Figure 3(A1,A2,B1,B2,C)).

We plotted RIBEYE A-domain puncta and RIBEYE B-domain puncta inside and outside of the synaptic ribbon in our post-embedding approach (Figure 3D). The number of RIBEYE A-domain and RIBEYE B-domain puncta within the synaptic ribbon was significantly higher than the RIBEYE A-domain and RIBEYE B-domain puncta outside of the synaptic ribbon (Figure 3D). Both antibodies were previously verified for their specificity with tissue from RIBEYE knockout mice [15,19], and RIBEYE is highly enriched at the synaptic ribbon [7]. Therefore, this finding was expected.

**Figure 4:**
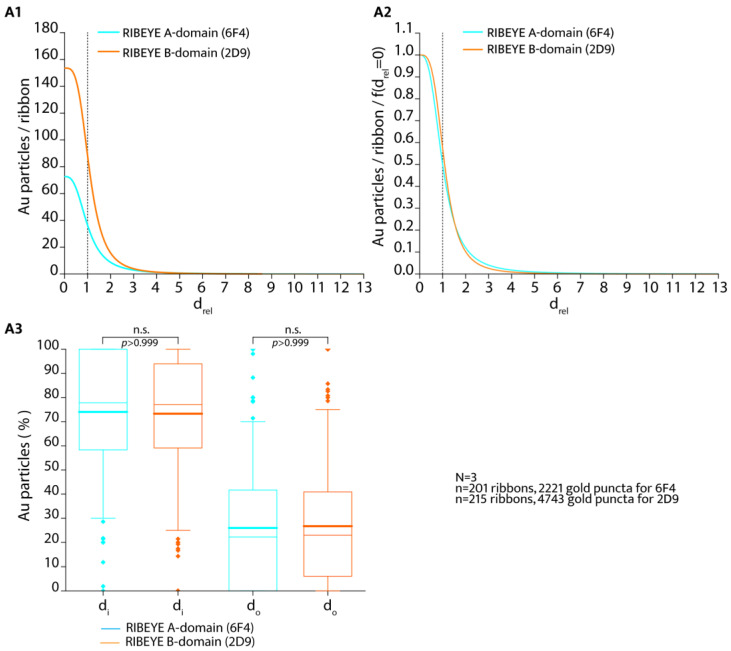
In (**A1**), only the fit curves for the RIBEYE A-domain and RIBEYE B-domain of the data presented in Figure 3(A1,A2,B1,B2) are shown (without depicting the individual data that are shown in Figure 3(A1,A2,B1,B2)). Curve parameters are given in the legend of Figure 3. In (**A2**), the curves from (**A1**) are normalized to the values of f(d_rel_ = 0) (i.e., at the midline of the synaptic ribbon) to correct for differences in the affinity of the antibodies that lead to different immunolabelling intensities. The dotted lines in (**A1,A2**) separate immunogold puncta with d_rel_ values ≤ 1 from immunogold puncta with d_rel_ > 1. In (**A3**), the relative values (percentage) of RIBEYE A-domain puncta and RIBEYE B-domain puncta located within the synaptic ribbon (d_i_) and outside of the synaptic ribbon (d_o_) were plotted. Boxes in (**A3**) represent the 25th to 75th percentiles of datapoints (interquartile range (Q1–Q3)), the lower whisker the 0.05-quantile (5% quantile) and the upper whisker the 0.95-quantile (95% quantile). Lower diamonds are datapoints smaller than the 0.05-quantile and upper diamonds are datapoints larger than the 0.95-quantile. Thick lines in (**A3**) depict the arithmetic mean, and thin lines represent the median value.

The quantitative analyses of the number of immunogold puncta revealed that the overall global labelling density of the synaptic ribbon differed between the two antibodies against the RIBEYE A-domain and RIBEYE B-domain (Figure 3(A1,A2) vs. Figure 3(B1,B2)). The overall labelling density of the synaptic ribbons was higher with the monoclonal antibody against the RIBEYE B-domain than with the RIBEYE A-domain (Figure 3). The RIBEYE A-domain and RIBEYE B-domain are present in RIBEYE in a 1:1 ratio. Different immunolabelling intensities could be based on different affinities of the antibodies (i.e., 2D9 and 6F4) for their respective epitopes. It is not surprising that antibodies differ in their binding affinities and typically have different binding affinities, often by several orders of magnitude [20,21,22,23,24]. If data were normalized to f(d_rel_ = 0) (density of immunogold puncta at the midline/centre of the synaptic ribbon), the distribution of RIBEYE A-domain puncta appeared very similar to the distribution of RIBEYE B-domain puncta (Figure 4(A2)).

We also plotted the percentage of RIBEYE A-domain immunogold particles and RIBEYE B-domain immunogold particles that were inside, i.e., within the outlines of the synaptic ribbon and outside of the synaptic ribbon (Figure 4(A3)). We did not find a significant difference in this comparison (Figure 4(A3)). An equal fraction of both RIBEYE A-puncta and RIBEYE B-puncta were found on/within the synaptic ribbon (arithmetic mean value of 74.0% for RIBEYE A-domain and 73.3% for RIBEYE B-domain; Figure 4(A3)). There was no significant difference between RIBEYE A-domain and RIBEYE B-domain distribution in these comparisons (Figure 4A3; *p* > 0.999; Kruskal–Wallis-ANOVA). Less than 30% of the RIBEYE A-domain- and RIBEYE B-domain puncta were outside of the synaptic ribbon for both the RIBEYE A-domain and RIBEYE B-domain. Again, these values were not statistically different from each other (*p* > 0.999; Kruskal–Wallis-ANOVA) (Figure 4(A3)).

**Figure 5:**
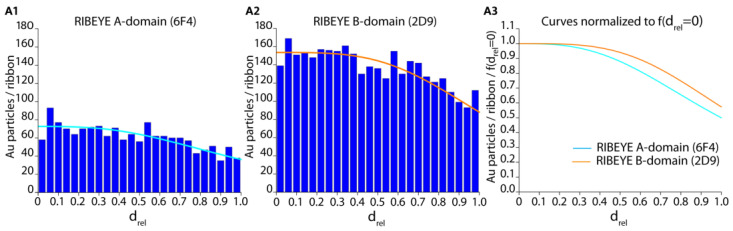
In Figure 5, only the relative distance (d_rel_) data with values between 0 and 1 (data from Figure 3) were plotted to analyze their frequency distribution on the synaptic ribbon at a higher resolution. Curve equation in (**A1**,**A2**) is the same as given in the legend in Figure 3 because only the area between d_rel_ = 0 (midline of the synaptic ribbon) and d_rel_ = 1 (surface of the synaptic ribbon) is zoomed in. In (**A3**) curves were normalized to f(d_rel_ = 0) to compensate for overall differences in immunolabelling densities. The data in (**A3**) do not differ significantly from each other, i.e., between RIBEYE A-domain and RIBEYE B-domain (*p* > 0.999; Kruskal–Wallis-ANOVA).

Most of the immunogold puncta, both for RIBEYE A-domain and RIBEYE B-domain, are located within the synaptic ribbon in post-embedding immunogold labelling with the gold-labelled primary antibodies (d_rel_ values between 0 and 1) (Figure 3(A1,A2,B1,B2); Figure 4(A1–A3)). Since it was hypothesized as a working model for the assembly of the synaptic ribbon that the RIBEYE A-domain might be located more toward the centre of the ribbon, whereas the RIBEYE B-domain could be positioned on the surface of the synaptic ribbon [7], we further analyzed the frequency distribution between d_rel_ = 0 (midline of the ribbon) and d_rel_ = 1 (surface of the synaptic ribbon) at a higher resolution (Figure 5) to obtain insights whether a differential gradient between RIBEYE A-domain and RIBEYE B-domain distribution could be observed.

The density of immunogold puncta was the highest in the centre of the synaptic ribbon (d_rel_ values from 0 to ~0.3; Figure 5(A1–A3)) and gradually declined towards the surface of the ribbon (at d_rel_ = 1) both for the RIBEYE A-domain and RIBEYE B-domain immunogold puncta (Figure 5(A1,A2)). As already mentioned above (Figure 3(A1,A2,B1,B2) and Figure 4(A1–A3)), the overall labelling density of the immunogold puncta within the synaptic ribbon for RIBEYE A-domain was less than for RIBEYE B-domain, as also judged by these high-resolution analyses (Figure 5(A1,A2)). If the relative distance values f(d_rel_) were normalized to f(d_rel_ = 0) (i.e., the value at the midline of the synaptic ribbon) (Figure 5(A3)), we observed a very similar distribution of the immunogold puncta for the RIBEYE A-domain and RIBEYE B-domain. For both the RIBEYE A-domain and RIBEYE B-domain, most of the immunogold particles were present in the inner third of the synaptic ribbon (Figure 5(A3)). Towards the surface of the synaptic ribbon (d_rel_ = 1), the labelling intensity slightly decreased for both antibodies (Figure 5(A3)).

In conclusion, we found immunogold puncta of the RIBEYE A-domain and RIBEYE B-domain in virtually the same relative distances to the centre of the synaptic ribbon without significant differences in the post-embedding immunogold labelling experiments with direct gold-labelled primary antibodies against the RIBEYE A-domain and RIBEYE B-domain. This finding suggests a refined model of the assembly of the synaptic ribbon (see Section 3).

## 3. Discussion

RIBEYE is the main and unique structural component of synaptic ribbons [7,8,9,13,14,15,16,25]. The deletion of RIBEYE leads to the complete absence of synaptic ribbons in the retina and inner ear. Other proteins are also found enriched at the synaptic ribbon (e.g., Piccolo/Piccolino; Bassoon, KIF3A ([26,27,28,29,30,31,32,33,34,35,36,37]; for review, see [3,38]). However, the deletion of these latter components does not lead to the disappearance of synaptic ribbons, suggesting that the RIBEYE protein forms the central building block of synaptic ribbons and cannot be substituted by other proteins. The self-assembly of RIBEYE subunits via RIBEYE–RIBEYE interactions might play an important role [17].

However, it is not yet clear how the ribbon is assembled from individual RIBEYE subunits. As mentioned in the introduction, RIBEYE has a conspicuous protein domain composition. The A-domain of RIBEYE is unique to RIBEYE, whereas the B-domain is virtually identical to CtBP2, a ubiquitously expressed transcriptional regulatory protein [7,10,11]. Since synaptic ribbons are only formed in ribbon synapses and since RIBEYE A-domain is the only protein domain of RIBEYE unique for RIBEYE, it was suggested that the RIBEYE A-domain is essential for the assembly of the synaptic ribbon [7]. Analyses of RIBEYE knockout mice in which the RIBEYE A-domain-encoding exon was deleted [9,13,14], together with data from cell transfection experiments [7,17], are compatible with this hypothesis. Recently, it was also shown that the B-domain of RIBEYE plays an important role in the assembly of the synaptic ribbon [15]. The replacement of the B-domain by another unrelated protein also abolished the formation of the synaptic ribbon [15].

It is incompletely understood how the synaptic ribbon is assembled from RIBEYE subunits and where exactly RIBEYE A-domain and RIBEYE B-domain are located within the synaptic ribbon in relation to each other. In the present study, we aimed to obtain further insights into this question by employing post-embedding immunogold electron microscopy with direct gold-labelled primary antibodies against the RIBEYE A-domain and RIBEYE B-domain for improved ultrastructural resolution. The monoclonal antibody against RIBEYE B-domain 2D9, in principle, can also detect CtBP2 because the corresponding epitope is shared between the CtBP2 and RIBEYE B-domain [19]. However, CtBP2 does not appear to be a quantitatively significant component of synaptic ribbons, as judged by Western blot analyses of purified synaptic ribbons [39]. Therefore, the immunolocalization data obtained with RIBEYE B-domain antibody 2D9 at the synaptic ribbon most likely reflect the localization of the RIBEYE B-domain on the synaptic ribbon but not CtBP2, which appears to be largely absent from the synaptic ribbon [39].

The antibodies against the RIBEYE A-domain (6F4) and RIBEYE B-domain (2D9) are antibodies of the IgG class of immunoglobulins. The size of IgG antibodies has been measured through the use of atomic force microscopy and determined to be 8.5 nm × 14.5 nm × 4.0 nm in its dimensions [40,41].

**Figure 6:**
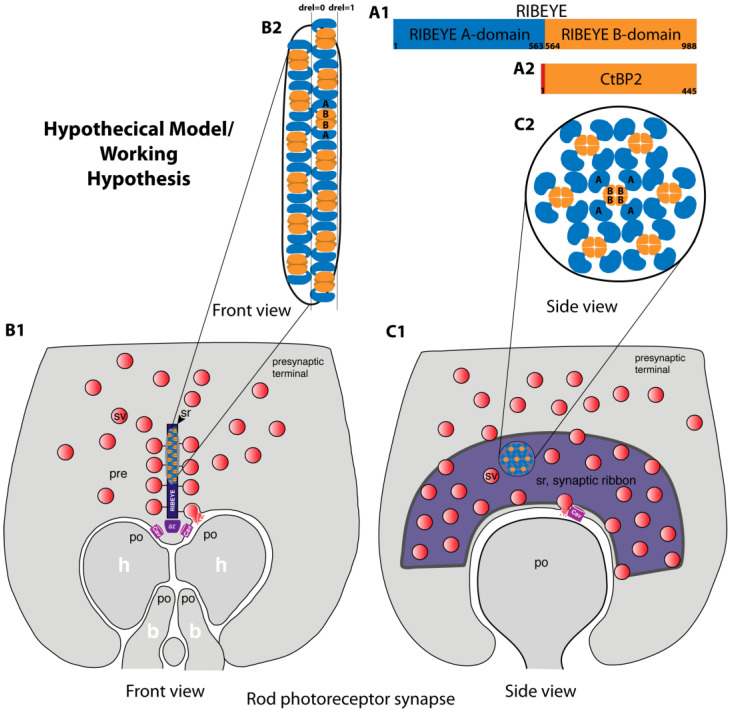
Hypothetical model**/**working hypothesis. (**A1**) Schematic cartoon of the protein domains of RIBEYE, the main protein component of the synaptic ribbon. The unique A-domain of RIBEYE is depicted in blue; the C-terminal RIBEYE B-domain, which is nearly identical to CtBP2 (**A2**), except for the first 20 amino-terminal amino acids, shown in orange. The numbers within the respective proteins indicate amino acid numbers. (**B**,**C**) schematically show a rod photoreceptor ribbon synapse in a front view (**B1**) and side view (**C1**). The schematic drawing of the principal components of a photoreceptor synapse in (**B1**,**C1**) is based on a drawing by [50]. In (**B2**,**C2**), the indicated regions of the synaptic ribbon are schematically magnified to depict the proposed details of our working model. The model is based on the assumption of a regular array of RIBEYE proteins within the synaptic ribbon. It summarizes our findings that the RIBEYE A-domain and RIBEYE B-domain are located at similar relative distances with reference to the midline of the synaptic ribbon. Please note that the molecular orientation of the RIBEYE molecules in (**B2**,**C2**), e.g., the schematically depicted localization of the N- and C-terminus of RIBEYE, is hypothetical and needs to be investigated in future structural analyses. The bold letters label the schematically depicted RIBEYE A-domain, annotated as A in (**B2**,**C2**), and RIBEYE B-domain, annotated as B in (**B2**,**C2**). Abbreviations: d_rel_ = 0, midline of the synaptic ribbon; d_rel_ = 1, outer border of the synaptic ribbon; sr, synaptic ribbon; sv, synaptic vesicle; Cav, voltage-gated calcium channels; az, active zone; pre, presynaptic terminal; po, postsynaptic dendrites; h, horizontal cell dendrite; b, bipolar cell dendrite.

As mentioned, RIBEYE B-domain is identical to CtBP2, except for the first 20 amino-terminal amino acids of CtBP2, which are absent from RIBEYE. The structure of CtBP2 has been solved [42,43,44,45,46,47,48,49]. CtBP2 (and the highly homologous CtBP1) form tetrameric complexes [45,46,47,48,49]. Initially, CtBP proteins have been characterized as dimeric proteins [42,43], but more recently it became clear that they predominantly form tetramers [45,46,47,48,49]. Tetramer formation is promoted by the binding of NAD(H) to CtBPs. Remarkably, tetramerization also occurs with N-truncated versions (CtBP_31-445_/CtBP_31-364_) that lack the N-terminal 30 amino acids of CtBP2 [46,47]. The RIBEYE B-domain lacks the first 20 N-terminal amino acids of CtBP2. Therefore, it appears reasonable to assume that RIBEYE B-domain also forms tetramers within the synaptic ribbon if enough NAD(H) is present, as schematically depicted in the schematic working model in Figure 6. The three-dimensional structure of the RIBEYE A-domain is still unclear.

The CtBP2 tetramer is roughly ~7 nm to ~9 nm in dimension (6wkw.pdb) and thus has similar size dimensions as the RIBEYE IgG antibodies 2D9 and 6F4, as mentioned above. Taking these size dimensions into consideration, we would expect to see a labelling gradient of immunogold particles on the synaptic ribbon in post-embedding immunogold electron microscopy with the direct gold-labelled primary RIBEYE antibodies if the RIBEYE B-domain is located preferentially at the outer surface of the ribbon and RIBEYE A-domain in the centre close to the midline of the synaptic ribbon. We did not see such a gradient, i.e., RIBEYE A-domain immunogold puncta preferentially at the midline of the synaptic ribbon and RIBEYE B-domain puncta close to the surface of the synaptic ribbon, with our direct immunogold labelling approach.

Instead, the RIBEYE A-domain puncta and RIBEYE B-domain puncta were similarly distributed within the synaptic ribbon (Figure 5). RIBEYE A-domain immunogold puncta and RIBEYE B-domain immunogold puncta displayed very similar distances from the midline of the synaptic ribbon (Figure 5). These data suggest a modified model on the architecture of the synaptic ribbon with RIBEYE A-domain and RIBEYE B-domain being located at similar relative distances with refer to the midline of the synaptic ribbon, as schematically summarized in our hypothetical working model shown in Figure 6.

The working model in Figure 6 also includes published structural data that demonstrated tetramerization of CtBP proteins in the presence of NAD(H) [45,46,47,48,49]. It also takes into account the previously identified RIBEYE-RIBEYE interaction sites in RIBEYE A-domain [17], which could give rise to multiple contacts between RIBEYE A-domains in Figure 6(B2,C2). At least three RIBEYE interaction sites were identified in the RIBEYE A-domain that could serve as a docking site for other RIBEYE A-domains [17]. These interaction sites could be important in generating a three-dimensional scaffold of the synaptic ribbon [17]. Whether these interaction sites are all engaged at the same time in the assembled synaptic ribbon or whether they mediate dynamic aspects of synaptic ribbon function remains to be elucidated by future investigations. Figure 6 represents a simplified working model that needs to be further refined by structural analyses (see below). Details of the model depicted in Figure 6, e.g., the molecular orientation of RIBEYE proteins with their schematically depicted N- and C-terminus, are hypothetical. Clearly, our morphological analyses cannot replace future structural analyses that target the atomic level of resolution of RIBEYE and of the assembled synaptic ribbons (see below).

The model in Figure 6 is based on the assumption of a dense and regular array of RIBEYE proteins within the synaptic ribbon. A regular array of particles has been previously observed through the use of freeze–fracture analyses of rod photoreceptor synaptic ribbons [47]. Previous freeze–fracture analyses showed a regular pattern of globular structures, roughly 10 nm in size, on the surface of photoreceptor synaptic ribbons [51]. These globular structures could represent individual RIBEYE proteins. The individual RIBEYE proteins could interact with each other via the previously identified RIBEYE–RIBEYE interaction sites present in the RIBEYE A-domain and RIBEYE B-domain [17] to provide the observed dense regular array of components.

As mentioned, RIBEYE A-domain and RIBEYE B-domain immunogold puncta showed very similar distances from the midline of the synaptic ribbon, and both domains might reach the surface of the synaptic ribbon, as schematically depicted in the simplified hypothetical model in Figure 6. At the surface the synaptic ribbon, both RIBEYE A-domain and RIBEYE B-domain could possibly bind to RIBEYE-interacting proteins. RIBEYE-interacting proteins are not included in Figure 6 for the sake of clarity. RIBEYE-interacting proteins include Bassoon, Piccolo/Piccolino, Tulp1, and others (for review, see [3]). Bassoon and Piccolo/Piccolino contain PXDLS motifs for binding to the RIBEYE B-domain (for review, see [3]).

### Limitations of the Study and Outlook

In our study, we used well-characterized mouse monoclonal primary antibodies against distinct epitopes of the RIBEYE A-domain and RIBEYE B-domain [15,19]. These IgG monoclonal antibodies were directly conjugated to gold particles with a size of 5 nm. This led us to propose a refined model of ribbon assembly and ribbon architecture presented as a working model in Figure 6. Direct immunogold labelling has a better spatial resolution than the standard indirect immunogold labelling technique. However, clearly, immunolabelling with direct labelled primary antibodies also has its resolution limits. The colloidal gold could, for example, be bound to the Fc region of the antibody some distance from the antigen-binding region of the antibody. To further improve the ultrastructural resolution of the localization of RIBEYE protein domains within the synaptic ribbon and to further refine the assembly model of the synaptic ribbon, one could use even smaller gold particles (e.g., ~1 nm “ultrasmall” gold particles), smaller antibody fragments (Fab fragments) directly labelled with gold particles, or alternative tags and freeze–fracture replica immunolabelling techniques (e.g., [52,53,54,55,56]). Exciting new super-resolution light microscopical techniques that were reported to achieve a resolution close to 1 nm [57] could also help to obtain closer insight into the molecular architecture of the synaptic ribbon. Structural investigations, e.g., using high-resolution cryo-electron microscopy of native vitrified samples/vitrified sections [58], structural analyses of RIBEYE A-domain and of full-length RIBEYE protein and of synaptic ribbons and related sub-molecular techniques, will be able to provide high-resolution details on the molecular anatomy of synaptic ribbons and its major component RIBEYE at the atomic level.

In the inner ear, synaptic ribbons are mostly spherical in shape [3]. The rod photoreceptor synaptic ribbons that we analyzed in the present study are bar-shaped (if cross-sectioned) or plate-shaped (in a side view) (Figure 6). It will be interesting to analyze whether the architecture in the spherical synaptic ribbons in the inner ear (in cochlear and vestibular hair cells) is similarly organized as in the rod–plate-shaped synaptic ribbons in rod photoreceptor ribbon synapses. Similarly, synaptic ribbons in cone photoreceptors and in retinal bipolar cells also have distinct size and shape characteristics. These might be based on a differential organization of the RIBEYE A-domain and RIBEYE B-domain within the synaptic ribbon or on differentially associated synaptic proteins.

## 4. Materials and Methods

### 4.1. Materials

#### Mice

All of the animal procedures (animal care procedures, anesthesia, and sacrificing mice for organ collection) were reviewed and approved by the local animal authorities (Tierschutzbeauftragte der Universität des Saarlandes and Landesamt für Verbraucherschutz; Geschäftsbereich 3; 66115 Saarbrücken, Germany; GB 3-2.4.2.2-25-2020). Mice were kept on a 10 h light–14 h dark cycle and provided with standard food and water ad libitum.

### 4.2. Primary Antibodies

#### RIBEYE Primary Antibodies

In the present study, we used two mouse monoclonal antibodies against the RIBEYE A-domain (6F4) and RIBEYE B-domain (2D9) for direct immunogold electron microscopy. These two RIBEYE antibodies have been previously shown to be specific for RIBEYE using tissue from RIBEYE knockout mice [15,19]. Both antibodies were previously successfully used for indirect immunogold electron microscopy to specifically detect RIBEYE in retinal ribbon synapses [15,19].

The anti-RIBEYE A-domain mouse monoclonal (clone 6F4, IgG1) was raised against aa83-211 of mouse RIBEYE [15]. Within this peptide region, the epitope of the 6F4 monoclonal RIBEYE A-domain antibody was previously further mapped to bind to the peptide stretch TGHLYPESGGKTVPHGQRTHGRAPSP (aa153-aa178 of mouse RIBEYE A-domain; [15]. This epitope is located within the “A3” interaction region of the RIBEYE A-domain that is located roughly in the middle portion of the RIBEYE A-domain (aa105-aa363; [17]).

The anti-RIBEYE B-domain mouse monoclonal antibody (clone 2D9, IgG2b) was raised against the last 12 carboxy-terminal amino acids (KHGDNREHPNEQ; aa977-aa988) of mouse RIBEYE [15,19]. This carboxy-terminal peptide stretch is highly conserved between species [7,19].

### 4.3. Methods

#### 4.3.1. Affinity-Purification of Mouse Monoclonal Antibodies from (NH_4_)_2_SO_4_-Precipitated Cell Culture Supernatant

Cell culture supernatants were collected from the respective hybridoma cells that were cultured in DMEM supplemented with 10% fetal calf serum (FCS) and 100 U/mL Penicillin/0.1 mg/mL Streptomycin (Sigma, Taufkirchen, Germany; P4333). Antibodies from the cell culture supernatants were precipitated with a saturated solution of ammonium sulphate precipitation (final (NH_4_)_2_SO_4_ 50%, *v*/*v*), as previously described [15,19,59]. (NH_4_)_2_SO_4_-precipitated antibody was dialyzed with Snake Skin dialysis tubing (10K-MWCO; Thermo Fisher, Dreieich, Germany, #68100) against a large excess of PBS (ON, 4 °C). The dialyzed antibody was affinity-purified with protein A-sepharose (Sigma, P3391-1), as previously described [60], to remove non-immunoglobulin contaminants. For this purpose, 200 μL–300 μL of (NH_4_)_2_SO_4_-precipitated antibody was added to a volume of 250 μL–500 μL protein A-sepharose suspension in PBS and incubated ON at 4 °C on an overhead rotary shaker. Unbound material was removed by several washes with ice-cold PBS. The bound antibody was released from the protein A sepharose beads by treatment with 200 µL of 0.2 M glycine, pH 2.7 (5 min, 4 °C, with gentle agitation in an overhead rotator). Afterwards, the suspension was briefly spun in an Eppendorf centrifuge (13,000 rpm, 1 min, 4 °C), and the supernatant with the eluted antibody was carefully removed. The pH of the supernatant was rapidly neutralized via the addition of 1 M Tris, pH 8.5, (typically 9–10 μL) to obtain a pH of 7.4. The antibody purity was verified using SDS-PAGE to contain only immunoglobulins (only immunoglobulin heavy and light chains) and the concentration of the eluted antibody was determined with a Nanodrop One Spectrophotometer (Thermo Fisher, Dreieich, Germany). The functionality of the affinity-purified antibodies was verified via indirect immunogold electron microscopy, performed as described before [15,19].

#### 4.3.2. Coupling of Affinity-Purified Monoclonal RIBEYE Antibodies to 5 nm Colloidal Gold

Absorption of antibodies to colloidal gold was performed as previously described [61,62,63]. For direct coupling of primary mouse monoclonal RIBEYE antibodies 2D9 and 6F4 to colloidal gold, the affinity-purified RIBEYE antibodies were dialyzed against 1 L of 2 mM borate buffer, pH 9.0, with Snake Skin dialysis tubing (10 K-MWCO; Thermo Fisher, Dreieich, Germany, #68100), as described above. Then, 5 μg of anti-RIBEYE B-domain antibody 2D9 and 5 μg of anti-RIBEYE A-domain antibody 6F4 (in a total volume of 20–40 μL) were added to 50 μL of 5 nm colloidal gold solution (BBI Solutions International, Cardiff CF10 3GA, UK, EM.GC5) and incubated for 1 hr for absorption of the antibody to colloidal gold particles (at 4 °C in an overhead rotator). The gold-conjugated primary antibodies were stored at 4 °C under constant agitation in an overhead rotator to avoid clustering of the colloid gold.

#### 4.3.3. Processing of Retinas for Post-Embedding Immunogold Electron Microscopy

The mouse retinas used in all experiments were acquired from adult C57BL/6J mice of either sex (female and male mice). The mice were killed in the early afternoon, and the eyes were collected under environmental daylight conditions. The posterior eyecup was dissected within 5 min post-mortem, as previously described [7,64,65].

The posterior eyecup with the attached retina was fixed in 2% freshly depolymerized paraformaldehyde (PFA) and 0.1% glutaraldehyde in PBS (~3 h, 4 °C). After several washes with PBS, the samples were treated with 0.1% tannic acid in PBS (1 h, 4 °C) and subsequently first washed with PBS and then 50 mM maleate buffer (pH 5.0). Next, the samples were treated with 2% uranyl acetate in maleate buffer (2 h, 4 °C). Afterwards, the samples were washed with maleate buffer and H_2_O and dehydrated in an ascending concentration series of pre-cooled ethanol solutions (30%, 50%, equilibrated to 4 °C; 70%, 80%, 90%, 99% (2×); pre-equilibrated to −20 °C; 15 min each). The samples were next infiltrated with increasing concentrations of LR Gold resin (LR-Gold/ethanol: 1:3, 1:1, 3:1 mixtures, 1 h each at −20 °C) before being transferred to pure LR Gold. LR Gold infiltration was performed overnight on an overhead rotator to promote infiltration of the LR-Gold resin. On the next day, the LR Gold was replaced with LR Gold containing 0.1% benzil and the samples were infiltrated for ~2 h on an overhead rotator. The samples were polymerized under UV light for ~2 days at −20 °C.

#### 4.3.4. Post-Embedding Immunogold Electron Microscopy with Directly Labelled Primary Antibodies

Ultrathin sections (70 nm in thickness) were made from the LR Gold-embedded retina samples with an ultramicrotome (Reichert–Jung). The ultrathin sections were incubated with 0.5% BSA in PBS (blocking solution) for 45 min at RT to saturate non-specific binding sites. Next, the sections were incubated with the gold-labelled primary antibody solutions. Gold-labelled anti-RIBEYE B-domain monoclonal antibody 2D9 was diluted at a ratio of 1:300 in blocking solution; Gold-labelled anti-RIBEYE A-domain antibody 6F4 was diluted at a ratio of 1:50 in blocking solution. Incubations were performed overnight at 4 °C. On the next days, the sections were washed with PBS to remove unbound primary antibodies. Immune complexes were fixed with 2.5% glutaraldehyde in PBS. After several washes with PBS and subsequently with H_2_O, the sections were contrasted with 2% uranyl-acetate in H_2_O for 15 min at RT. The sections were analyzed with a Tecnai Biotwin 12 transmission electron microscope (FEI/ThermoFisher, Eindhoven, The Netherlands) equipped with a Megaview III digital camera (Gatan, Unterschleissheim, Germany) that was controlled using iTEM acquisition software version 5.0 (Olympus, Hamburg, Germany).

Please note that post-embedding immunogold immunolabelling is a surface labelling procedure that labels antigens that are exposed on the surface of the LR Gold-embedded resin sections.

#### 4.3.5. Conventional, Indirect Post-Embedding Immunogold Electron Microscopy

Conventional, indirect immunogold electron microscopy with gold-labelled secondary antibodies was performed as a positive control to show the functionality of the affinity-purified secondary antibody. Post-embedding immunogold microscopy with the indirect method was performed exactly as previously described [7,64,65]. For the indirect immunogold labelling procedure, the primary affinity-purified, unconjugated RIBEYE antibodies 2D9 and 6F4 were used in a 1:200 dilution in blocking solution. For the secondary antibody, goat-anti mouse immunoglobulins were used that were conjugated to 5 nm gold particles (Sigma, Taufkirchen, Germany; G7527) at a 1:100 dilution in blocking buffer (1 h, RT).

#### 4.3.6. Topographical Analysis of Ribbon-Bound Immunogold Particles on the TEM Images of Photoreceptor Synaptic Ribbons

For the quantitative analyses, we used three independent embeddings from three different mice that were immunolabelled with the gold-conjugated primary RIBEYE antibodies, as described above. For each antibody, three immunolabelled grids were generated from each mouse retina embedding in three different experiments. For the topographic analyses of the localization of the immunogold particles with reference to the synaptic ribbons, we acquired TEM images at a primary magnification of 135,000×. Analyses were performed blindly, i.e., the experimenter did not know whether the respective section was obtained from immunolabelling with the antibody against the RIBEYE A-domain or RIBEYE B-domain.

For topographical analyses, images were opened with Adobe Photoshop CS6 (Adobe Systems Incorporated, San Jose, CA, USA), and the electron-dense area of the synaptic ribbon was marked manually. The marked area was surrounded by a line to demarcate the outer border of the synaptic ribbon. Next, the digital ruler in Photoshop was used to mark 5–20 midpoints between the borders of the ribbon along its entire length. The number of the midpoints was adjusted to the size and curvature of the synaptic ribbon. The midpoints were connected from the midline (centre) of the synaptic ribbon. This line was used as a reference to determine the relative localization of the immunogold puncta (see below). Each RIBEYE immunogold punctum was marked manually by placing a coloured dot in the middle of the gold particle (Figure 2C). For the subsequent automated topographical analyses with a Python-based, self-made programme, immunogold puncta inside the outline of the synaptic ribbon were labelled in one colour; RIBEYE immunogold puncta outside the outlines of the synaptic ribbon (i.e., on the surface of the labelled electron-dense structure of the synaptic ribbon) were labelled in another colour (Figure 2C). This was carried out because our self-programmed Python-based analysis software determines the relative position of the RIBEYE puncta inside or at the border of the synaptic ribbon and the RIBEYE puncta outside of the synaptic ribbon via different equations (see explanation below). Therefore, RIBEYE immunogold puncta within the synaptic ribbon and outside of the ribbon needed to be marked differently for the subsequent automated analysis by the analysis software. The Python-based analytical software autonomously analyzed the coordinates of the outer border, the midline, and the position of the marked immunogold particles.

For RIBEYE immunogold puncta that were located within the electron-dense borders of the synaptic ribbon, the relative position of RIBEYE puncta with reference to the midline of the synaptic ribbon was determined with the following Equation (1):

(1) $$d_{i}=\frac{d_{1}}{d_{1}+d_{2}}$$

d_1_ = minimal distance from the midline to the gold particle.
d_2_ = minimal distance from the gold particle to the outer border of the ribbon.
d_i_ = inner relative distance to the midline of the synaptic ribbon.

With Equation (1), the localization of the gold particles can range from 0 (midline of the ribbon) to 1 (border of the ribbon). To also analyze the gold particles outside of the ribbon and quantitatively compare these values between RIBEYE A-domain and RIBEYE B-domain, we used Equation (2):

(2) $$d_{o}=\frac{d_{1}}{d_{1}-d_{2}}$$

d_1_ = minimal distance from the midline to the gold particle.
d_2_ = minimal distance from the gold particle to the outer border of the ribbon.
d_o_ = outer relative distance with reference to the midline of the synaptic ribbon.

Immunogold puncta that were located outside of the synaptic ribbon were analyzed using Equation (2). In the case of Equation (2), gold puncta outside of the synaptic ribbon obtain values >1 graded due to their distance from the synaptic ribbon. The bigger the distance from the ribbon midline, the more the d_o_ value is larger than 1 as a relative distance to the sectioned ribbon surface. The values obtained with this equation are always bigger than 1 and increase in size the more distant the puncta are located away from the ribbon.

The relative distances of each antibody for each of the mice were plotted with OriginPro2018b as histograms, depicting the number of gold particles for each relative distance (d_rel_). The relative distances from zero to one (d_i_) represent the immunogold particles inside the area of the ribbon. Relative distances above one (d_o_) represent immunogold particles outside of the synaptic ribbon.

### 4.4. Statistical Analyses

The statistical analysis of the relative distance data obtained by the directly labelled antibodies against RIBEYE A-domain and RIBEYE B-domain was carried out with the help of OriginPro 2018b software (OriginLab Corporation 1991–2018, Northampton, MA, USA) and Prism 9 (GraphPad Software, Boston, MA, USA).

First, the raw relative distance data d_rel_ (i.e., d_i_ and d_o_) were plotted for both RIBEYE A-domain immunogold puncta (6F4) and RIBEYE B-domain immunogold puncta (2D9) with OriginPro. The data were fitted with a logistic fit using the following equation:

(3) $$f:\;[0,\;13]\;\to \;R,\;x\;\mapsto f\left(d_{rel}\right)=a_{2}+\frac{\left(a_{1}-a_{2}\right)}{1+{\left(\frac{d_{rel}}{d_{relcenter}}\right)}^{p}}$$

a_1_ = f(d_rel=0_); a_2_ = $\underset{d_{\mathrm{rel}}\to \infty }{\lim}f(d_{rel})$; p = power.

It is not unexpected that two different antibodies generate different immunolabelling densities in post-embedding immunogold microscopy because two different antibodies are unlikely to have the same affinity to their epitope. Furthermore, the respective epitopes could not be made accessible in an identical manner. To better compare the relative distribution of RIBEYE A-domain immunogold puncta (6F4 puncta) with RIBEYE B-domain immunogold puncta (2D9 puncta) and to better visualize potential differences in the localization of the RIBEYE A-domain and RIBEYE B-domain puncta, the data were normalized. For this purpose, the value at d_rel_ = 0 (value at the midline of the synaptic ribbon; see also Figure 2(B2,C)) was set to 100% for each antibody. The normalized values were also fitted with Equation (3).

For each antibody and each of its grids, the number of gold particles per ribbon in the interval d_i_ was compared to the number of gold particles per ribbon in the interval d_o_, and the antibodies were then compared to each other using the non-parametric Kruskal–Wallis test, followed by a post hoc Dunn’s test. The results were depicted in a box plot, showing the differences between the number of gold particles inside and outside the area of the ribbon. Also, the percentage of the number of particles inside (d_i_) and outside (d_o_) of the marked area compared to the number of all gold particles was calculated for each antibody and compared using Kruskal–Wallis and post hoc Dunn´s test. The results were also plotted as box plots to depict the individual datapoints.

## Data Availability

The original contributions presented in the study are included in the article, and further inquiries/requests can be directed to the authors.
